# Venovenous bypass in liver transplantation: Exploring the benefits, efficacy, and safety

**DOI:** 10.1051/ject/2024005

**Published:** 2024-06-18

**Authors:** Salman Pervaiz Butt, Arun Kumar, Fazil Ashiq, Andrei Minou, Giuseppe Iuppa, Cristiano Quintini

**Affiliations:** 1 Perfusionist & ECMO Specialist, Heart Vascular and Thoracic Institute, Cleveland Clinic PO Box: 112412 Abu Dhabi United Arab Emirates; 2 Department Chair, Cardiothoracic Aesthesia, Anesthesiology Institute, Cleveland Clinic Abu Dhabi United Arab Emirates; 3 Anesthesiology Physician, Anesthesiology Institute, Cleveland Clinic Abu Dhabi United Arab Emirates; 4 Anesthesiology Institute, Cleveland Clinic Abu Dhabi United Arab Emirates; 5 Digestive Disease Institute, Cleveland Clinic Abu Dhabi United Arab Emirates; 6 Institute Chair, Digestive Disease Institute, Cleveland Clinic Abu Dhabi United Arab Emirates

**Keywords:** Liver transplant, Venovenous bypass, Benefits

## Abstract

Venovenous bypass (VVB) is a technique used in liver transplantation (LT) to maintain hemodynamic stability and abdominal organ perfusion and thereby improve patient outcomes. Despite its perceived benefits, VVB utilization has declined globally due to concerns related to heparinization, major bleeding and the need for expertise. Recent advancements, such as percutaneous cannulation techniques and improved extracorporeal technology have improved the safety of VVB in LT. This paper presents a modified VVB circuit with enhanced safety features. Cannulation plays a pivotal role in VVB establishment, with percutaneous methods increasingly favored. Studies demonstrate VVB’s efficacy in improving patient outcomes with lower incidence of acute kidney injury and reduced operative time and blood loss, with no added morbidity or mortality. However, its routine use faces challenges, with alternative techniques gaining traction. Our experience highlights VVB’s role in various clinical scenarios, including patients with high Model for End-Stage Liver Disease (MELD) scores, challenging surgical anatomy, portal vein thrombosis and pre-existing cardiovascular disease, emphasizing its safety and efficacy. Continued research is needed to optimize VVB techniques and ensure better outcomes for liver transplant recipients.

## Introduction

Venovenous bypass (VVB) is a technique employed during liver transplantation (LT) to redirect blood flow from Inferior vena cava (IVC) and portal circulation. This technique brings multiple advantages, notably minimizing the necessity for using the classic technique of IVC clamping. By diverting venous blood away from the liver, VVB maintains hemodynamic stability, prevents complications associated with prolonged clamping, and enhances recipient outcomes. Additionally, VVB shortens the anhepatic phase, reducing the risk of ischemia-reperfusion injury. Although the adoption of VVB requires ECC and specialized equipment, its integration into routine liver transplants holds great promise for improving outcomes and accessibility of this procedure [1–3].

The introduction of VVB in LT has a long history marked by significant milestones. Dating back to the early 1960s, initial attempts at employing ECC techniques during LT were made by Dr. Thomas Starzl. However, these early efforts, including heparinization and venoarterial bypass, were soon abandoned due to unsatisfactory outcomes. A breakthrough came in the early 1980s when the Pittsburgh group introduced the VVB method, which utilized specialized equipment and techniques. This innovative approach, characterized by heparin-bonded tubing and blood propulsion via a low-pressure vortex principle, offered improved perfusion of the venous system with reduced trauma to blood. Over time, advancements have been made in bypass systems, such as the utilization of Biomedicus centrifugal pumps and the introduction of the Griffith TDMAC Veno-venous Shunt. These milestones in the evolution of VVB techniques have significantly contributed to addressing challenges associated with heparinisation and major bleeding during LT. Additionally, the development of rapid transfusion systems, pioneered by the Pittsburgh group, has further improved the management of rapid blood loss during surgical procedures [4].

Despite being available and utilized since the 1980s, the routine use of VVB has experienced a decline in LT procedures worldwide. Several factors contribute to this trend, including the high cost of VVB, the associated risks of large-bore line insertion and the bypass procedure itself, the potential for hypothermia, and the availability of alternative techniques [3].

Over the past two decades, advancements in VVB techniques has taken place, including the emergence of a percutaneous technique as a safer and easier alternative to the traditional surgical cut-down method. This percutaneous approach has shown promise in terms of improved safety and ease of implementation. Furthermore, advancements in extracorporeal technologies including better design, incorporation of heat exchanger devices to prevent hypothermia, and availability of coated circuits for better anticoagulation management has expanded the utilization of VVB in critically ill patients undergoing LT, offering opportunities to enhance patient outcomes [1].

## Methods

**Figure 1:**
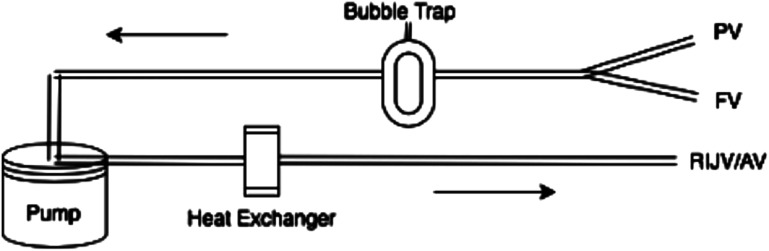
Illustration of veno-venous bypass for liver transplantation. FV – Femoral Vein, PV – Portal Vein, RIJV – Right Internal Jugular Vein, AV – Axillary Vein.

We present a modified VVB circuit that incorporates a heat exchanger, illustrated in Figure 1. This addition enhances the traditional method, which uses a centrifugal pump and a single access and return line, by offering precise temperature control to efficiently warm or cool the patient as needed. Additionally, the inclusion of bubble sensors on both the access and return lines ensures the safety of the circuit by promptly detecting and mitigating any potential risks associated with air embolism. The circuit used is also coated with heparin to reduce the need for anticoagulation and improve biocompatibility.

**Figure 2:**
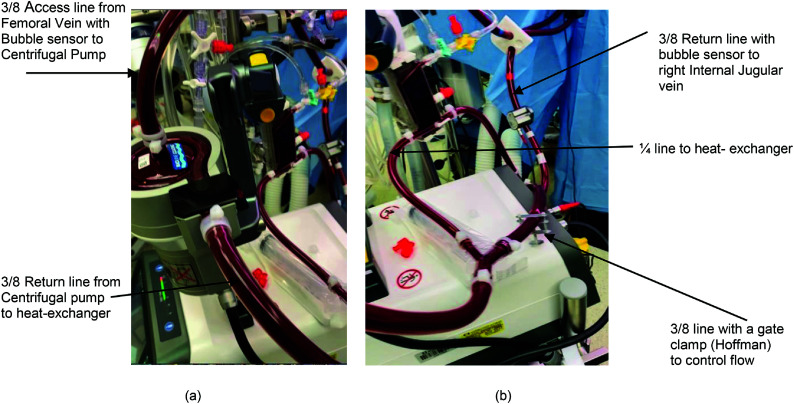
Illustration of venovenous bypass circuit. (a) Centrifugal pump with access and return line and a heat exchanger added to the return line. (b) Bypass loop with gate clamp (Hoffman) to control flow through the heat exchanger.

From a hardware standpoint, we utilize the Rotaflow II System machine (Getinge, Göteborg, Sweden) equipped with two bubble detectors and flow probes as illustrated in Figure 2. These components are affixed to the access and return lines of the circuit. The system offers configurable interventions, such as halting the pump in case air bubbles are detected in either line. Moreover, the console features a flow limits function enabling the establishment of lower and upper alarm thresholds. In addition, we employ the DLP 60000 pressure Display box (Medtronic, Minneapolis, Minnesota, USA) to monitor the overall circuit pressure, complete with adjustable lower and upper-pressure settings. Furthermore, temperature control is facilitated by the Heater Unit HU 35 (Getinge, Göteborg, Sweden).

From a circuit perspective, we employ the Rotaflow pump head coupled with tubing featuring a bioline coating as this enables the utilization of minimal anticoagulation doses during the procedures. Temperature regulation is managed through the Sorin CSC 14 Cardioplegia heat exchanger set (Livanova, London, United Kingdom). The access and return cannulas utilized are also coated with Bioline (HLS), with the preferred choice being 15 Fr femoral aortic for access and return, as the required flow typically falls between 1.8 and 2.2 L/min.

Moreover, while a hemoconcentrator can be incorporated into the VV bypass circuit if necessary, we opt not to include any additional devices in the circuit to minimize the risk of circuit air embolism. Instead, if required, we utilize continuous renal replacement therapy (CRRT) intraoperatively as a separate measure.

In terms of anticoagulation, our protocol entails administering a standard pre-VV bypass dose of 3000 units of heparin for all patients. Additionally, we prepare two bags of 500 mL of normal saline, with a concentration of 2 units per mL. These bags are connected to both the access and return cannulas, with infusion rates maintained at 1–2 mL per minute.

## Cannulation

**Figure 3:**
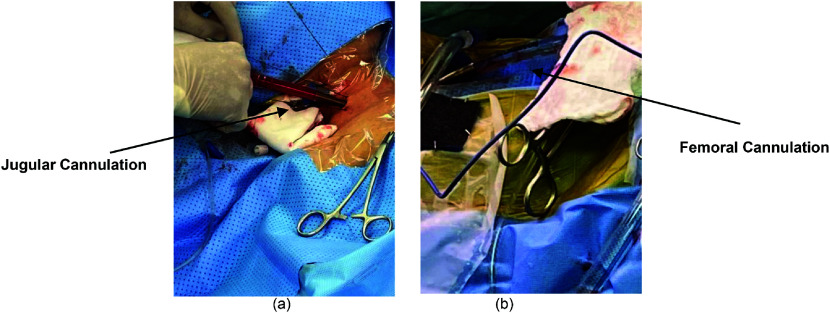
Illustration of jugular (a) and femoral (b) percutaneous cannulation.

Cannulation plays a pivotal role in the establishment of VVB. An 18G cannula is placed in the femoral vein for access by Anaesthesiologist. The drainage cannula is then inserted into the femoral vein by the surgeon, facilitating the drainage of blood below the infrahepatic inferior vena cava (IVC). The blood is returned to a 15Fr cannula placed in the internal jugular vein percutaneously by the Anesthesiologist via the bypass circuit as illustrated in Figure 3. If a portal bypass is necessary, a second drainage cannula is inserted into the portal vein and connected to the main VVB circuit through a dedicated drainage line. To ensure optimal functionality, it’s important to use high-volume, low-pressure inflow and outflow cannulas.

Catheter access can be achieved either percutaneously or, on rare occasions, through surgical cutdown. Surgeon performs the femoral or Portal cannulation and Anesthesiologists typically perform percutaneous internal jugular venous cannulation [5, 6].

## Discussion

Several studies have evaluated the use of the venovenous/portal (VVP) bypass technique in LT and found positive outcomes. The studies demonstrated that VVP bypass can offer benefits such as hemodynamic stability, prolonged surgical time, and improved outcomes for patients with renal disease. These findings support the reconsideration of the extracorporeal VVP bypass as a means to minimize complications and improve patient outcomes in LT procedures [2, 3].

VVB provides numerous advantages, such as sustaining stable hemodynamics during the transplant procedure and lessening the likelihood of complications like cardiac arrhythmias, pulmonary hypertension, and right ventricular dysfunction. Additionally, it enhances recipient safety by lowering the chances of surgical complications.

The efficacy of the venovenous/portal (VVP) bypass technique in LT was assessed through an analysis of 163 consecutive LTs conducted at a center since the inception of its liver transplant program in 2010. The average operative time was 269 min with a warm ischemic time of 43 min. Median transfusion requirements for packed cells and plasma were 7 and 14 units, respectively. No intraoperative deaths were reported, and 30-day mortality stood at 3%, with no severe bypass-induced complications observed. The discussion emphasizes the significance of stringent safety measures during the establishment of new LT programs, highlighting the precise control offered by the VVP bypass device over surgical and anesthesiological management, particularly beneficial when utilizing marginal grafts. This approach aims to minimize volume overload, reduce vasopressor usage, mitigate myocardial injury, and improve peripheral blood circulation. Consequently, based on the findings, there’s a suggestion for a reconsideration of the extracorporeal VVP bypass in LT [7].

In a comparative study, researchers investigated the impact of VVB during liver resections with prolonged hepatic vascular exclusion and hypothermic liver perfusion. They found that VVB use led to significantly reduced intraoperative blood loss (*p* = 0.010) and fewer postoperative respiratory complications (15% in patients with venovenous bypass VVB+ vs. 64% in patients without venovenous bypass VVB−, *p* = 0.012). Despite VVB+ patients experiencing longer operative times (460 vs. 375 min, *p* = 0.023), there were no significant differences in postoperative mortality or major morbidity rates between the VVB+ and VVB− groups. These results underscore the potential benefits of VVB in enhancing surgical outcomes during complex liver resections with prolonged hepatic vascular exclusion and hypothermic liver perfusion, emphasizing its recommendation in such cases [8].

Sakai et al. reported that the adoption of the retrohepatic caval preservation technique in LT has significantly reduced the need for VVB, marking a notable advancement in surgical methodology. However, VVB remains a valuable adjunct in LT procedures. Traditionally, the insertion of the venous return cannula via a cut-down technique through the axillary vein posed significant risks such as lymphorrhea, infection, or nerve damage. Since 2001, attending anesthesiologists have routinely performed percutaneous insertion of the internal jugular venous return cannula in adult liver transplant surgeries as part of their clinical practice, providing a safer alternative. This approach not only reduces associated risks but also enhances the overall safety and efficacy of the surgical intervention [9].

A retrospective study assessed VVB’s impact on post-liver transplant acute kidney injury (AKI). Among 1037 patients, 247 received VVB. AKI incidence was lower in VVB patients with pretransplant renal dysfunction (Cr ≥ 1.2 mg/dL), and VVB was independently associated with reduced AKI risk. No significant differences were observed in renal replacement or 1-year mortality. In patients with normal renal function (Cr < 1.2 mg/dL), AKI incidence did not differ between groups. This study suggests intraoperative VVB may mitigate posttransplant AKI risk in those with compromised renal function, necessitating further investigation [10].

A study by Rocco et al. presents two cases of complex orthotopic LT where VVB with the insertion of a venous graft into either the inferior mesenteric vein (IMV) or the splenic vein (SV) was utilized for decompression of the portomesenteric compartment. In both cases, femoroaxillary percutaneous VVB was established prior to abdominal opening to alleviate massive collateral veins in the abdominal wall. The first patient had the IMV connected to a donor vein graft, while the second patient required splenectomy due to an excessively enlarged spleen, with the SV connected to a donor vein graft. In both instances, connecting the distal part of the vein graft to the VVB facilitated decompression of the portomesenteric compartment, reducing portal hypertension and enabling access to the hepatic hilum for the intricate dissection necessitated by previous major surgeries. This technique demonstrates safety and simplicity, proving beneficial for patients requiring VVB without standard access to the portal compartment, especially in cases of severe portal hypertension and re-LTs [5].

A case report presents a pioneering method for VVB during LT utilizing a patent para-umbilical vein, a previously undocumented technique. In a patient necessitating VVB during LT, a pre-transplant CT scan identified a sizable patent para-umbilical vein. Prior to abdominal opening, a femoro-axillary percutaneous VVB was established, linking the para-umbilical vein to the VVB. This inventive approach effectively facilitated splanchnic venous decompression throughout the surgery. The utilization of the para-umbilical vein in VVB during LT signifies a promising avenue for similar cases in the future [11].

At our center, we employed VVB for LT in 11 patients with diverse indications outlined in Table 1, including High Model for End-Stage Liver Disease (MELD) scores, previous abdominal surgeries and adhesions, multiple spontaneous bacterial peritonitis, portal vein thrombosis, and coronary artery disease with heart failure.

**Table 1:** Indications for VV bypass utilization and indications.

Case	Indication
1	High MELD score
2	Previous abdominal surgery and adhesions
3	High MELD score
4	Multiple spontaneous bacterial peritonitis causing “cocoon abdomen”
5	Portal vein thrombosis
6	High MELD score
7	Extensive abdominal adhesions
8	Pre-existing cardiovascular disease
9	Portal vein thrombosis
10	High MELD score
11	Portal vein thrombosis

In our experience, none of the cases exhibited vascular complications associated with cannulation or issues related to VVB. Furthermore, post-reperfusion syndrome was not observed in any of the cases.

## Conclusion

The integration of VVB into routine liver transplant procedures requires expertise and specialized equipment. However, as more centers gain experience and refine their protocols, the widespread implementation of VVB holds great potential. Further research and clinical trials are necessary to refine techniques, explore long-term benefits, and ensure the seamless incorporation of VVB into the surgical armamentarium. In our experience, VVB is a valuable technique in LT that offers numerous benefits, including facilitating complex surgeries, maintaining hemodynamic stability, and enhancing safety for recipients. No vascular complications or VVB-related issues were observed in our cases, and there were no instances of post-reperfusion syndrome.

## Data Availability

The research data is available on request from the authors.
